# Maternal factors associate with microbiota-derived extracellular vesicle profiles in pregnancy: a clinical cohort study

**DOI:** 10.1186/s12916-026-04960-3

**Published:** 2026-05-28

**Authors:** Jenni Turunen, Kaisu Kyrö, Janica Virta, Marko Suokas, Jenni Hekkala, Sonja Karikka, Anna Kaisanlahti, Justus Reunanen, Niko Paalanne, Mysore V. Tejesvi, Terhi Ruuska-Loewald

**Affiliations:** 1 Research Unit of Clinical Medicine, University of Oulu, Oulu, Finland; 2 Biocenter Oulu, University of Oulu, Oulu, Finland; 3 Research Unit of Translational Medicine, University of Oulu, Oulu, Finland; 4 Laboratory of Developmental Biology, Disease Networks Research Unit, Faculty of Biochemistry and Molecular Medicine, University of Oulu, Oulu, Finland; 5 Kerttu Saalasti Institute, University of Oulu, Oulu, Finland; 6 Department of Pediatrics and Adolescent Medicine and Medical Research Center, Oulu University Hospital, Oulu, Finland

**Keywords:** bacterial extracellular vesicles, microbiota, gut, amniotic fluid, pregnancy, obesity, gestational diabetes, antibiotics, antimicrobial agents, fetal period

## Abstract

**Background:**

While most clinical microbiome research has investigated gut microbiota, less is known about extracellular vesicles (EVs) produced by microbiota. Microbiota-derived EVs form a distinct taxonomic entity from the gut microbiota. Recently, microbiota-derived EVs from maternal microbiota have been shown to reach the fetus, which could be an important mechanism of microbiota–host interaction during the fetal period. We hypothesized that maternal factors could change the composition of microbiota-derived EVs during pregnancy.

**Methods:**

We compared the influence of antibiotics and maternal weight on microbiota-derived EVs secreted by maternal microbiota during pregnancy. We collected fecal samples from 60 pregnant women and 18 amniotic fluid samples from those undergoing C-section. Microbiota-derived EVs were isolated from the samples using size-exclusion chromatography and density-gradient ultracentrifugation and characterized using transmission electron microscopy (TEM) and nanoparticle tracking analysis (NTA). RNA was isolated from the microbiota-derived EVs and converted to complementary DNA (cDNA), and 16 S rRNA gene sequencing was performed.

**Results:**

Altogether, 18 (30%) women had received antibiotics during pregnancy and 37 (62%) during delivery. Twenty (33%) women were lean, 27 (45%) had overweight and 13 had obesity (22%) during the first trimester. Taxonomic diversity of microbiota-secreted microbiota-derived EVs was lower in women exposed to antibiotics during pregnancy. When women exposed to antibiotics during delivery were excluded, the differences were not statistically significant. The microbiota-derived EVs differed in the amniotic fluid and maternal feces in women with overweight and obesity, gestational diabetes (GDM), and those who gained > 15 kg during pregnancy, as many bacterial origins of microbiota-derived EVs were depleted in these women. A beta diversity analysis of microbiota-derived EVs from fecal samples showed significant differences between the overweight and obesity groups. Diversity analysis showed no differences in various health factors during pregnancy, including asthma, allergies, or smoking.

**Conclusions:**

Maternal factors influence the composition of microbiota-derived extracellular vesicles in feces and amniotic fluid of pregnant women, which may change host-microbiota interaction in the fetal period.

**Supplementary Information:**

The online version contains supplementary material available at 10.1186/s12916-026-04960-3.

## Background

Microbiota-derived EVs are lipid-bilayer nanoparticles secreted by virtually all bacteria [1]. They are a functional layer of the microbiota, likely interacting with the host in various ways [2]. While most clinical microbiome research has investigated the gut microbiota, microbiota-derived EVs have been shown to form a distinct entity from the gut microbiota in various clinical cohorts, including pregnant women [3]. Microbiota-derived EVs cross biological barriers [4] and carry various biomolecular cargo, such as proteins, metabolites, and nucleic acids [4], to body compartments, even those traditionally considered sterile, such as the uterus [5, 6]. In a recent study, we have shown that both maternal gut microbiome and the fetal environment contain microbiota-derived EVs, and in a mouse model, maternal microbiota-derived EVs were found to reach the fetus *in utero* [7].

Maternal factors significantly affect the health of both the mother and the fetus during pregnancy. The use of antibiotics during pregnancy has been suggested to increase the risk of increased adiposity in children [8, 9] and different autoimmune diseases, such as allergy and atopy [10]. Obesity predisposes pregnant women, fetuses, and newborn infants to adverse pregnancy- and birth-related outcomes [11] and influences the gut microbiota and its functions during pregnancy [12]. However, the exact mechanisms by which maternal factor-related changes in the gut microbiota affect the developing fetus are not fully understood. We hypothesized that maternal factors change the secretion of microbiota-derived EVs from the maternal microbiota, which in turn could affect fetal development.

In the present study, we investigated how maternal factors influence the microbiota-derived EVs secreted by the maternal microbiota into the amniotic fluid and feces of pregnant women.

## Methods

### Study cohort and study design

This was a clinical cohort study of 508 mother–infant dyads investigating fetal and early-life factors and the microbiota. Pregnant women were recruited before an elective C-section or upon arrival at the hospital for vaginal delivery. All participants gave fecal samples, but amniotic fluid samples were obtained in a sterile manner only from those undergoing elective C-sections. For the present study on microbiota-derived EVs and the metabolic status of pregnant women, 60 fecal samples and 18 amniotic fluid samples were collected from 60 randomly selected women. The research plan was reviewed by the Ethics Committee of Oulu University Hospital, Finland, before the study, which issued decision number ETTMK:3/2016. The participants provided written informed consent before the study.

### Sample collection

An obstetrician collected amniotic fluid samples from the women during their elective C-sections in a sterile manner in the operation room. The samples were collected with a sterile needle inserted through the fetal membrane or collected first to a sterile kidney dish after opening the amniotic cavity in a sterile manner during C-section. None of the amniotic fluid samples were contaminated by first-pass meconium. The pregnant women gave fecal samples after recruitment in the hospital or at home after discharge. Home samples were promptly delivered to the hospital by mail. Fecal samples were sent in commercially used clinical fecal sample vials without buffer. The participants were given identical written sample collection and, in case of home collection samples, delivery instructions including photos of the equipment and vials. Each sample was promptly frozen, stored, and processed in a similar manner before the analysis.

### Isolation and characterization of extracellular vesicles

EV isolation and characterization were based on a protocol by Tulkens et al. [13]. and documented on EV-TRACK [14] (ID: EV250013) [15]. In total 232–1,138 mg of fecal sample was used for isolation. One sample had too little fecal material to be accurately weighed. Six negative controls consisting of sample-free Falcon tubes with 15 mL of phosphate-buffered saline (PBS, Sigma-Aldrich, St. Louis, MA, USA) were processed alongside the samples. The samples were first processed by filtering out non-EV material by centrifuging twice at 14,000 g for 30 min at 4 °C and filtering the supernatants using 40 μm nylon filters (Thermo Fisher Scientific, MA, USA) and 0.45 μm PES-filters (Thermo Fisher Scientific). The filtrates were concentrated using Amicon^®^ Ultra-15 Centrifugal filter units (100k) (Merck Millipore, MA, USA) according to the manufacturer’s guidelines, and EVs were isolated from the concentrates using Exo-Spin™ mini-columns (Cell Guidance Systems, Cambridge, UK) according to the manufacturer’s guidelines. Microbiota-derived EVs were separated from other particles in the samples with overnight density-gradient ultracentrifugation (18 h, 100,000 g, 4 °C). The density gradients were prepared using 5, 10, 20 and 40% OptiPrep™ gradient medium (Axis Shield, Bristol, UK) pipetted on top of each other, with the most diluted solution on top and least diluted on bottom. The sample was pipetted on top of the 5% solution. Finally, microbiota-derived EVs were purified by 2.5 h ultracentrifugation at 100,000 g/4°C. The used protocol was designed specifically for isolating microbiota-derived EVs with minimal human EV presence based on earlier validation by immuno-electron microscopy, western blotting, and ExoView using specific human markers [16].

Amniotic fluid samples were processed by mixing 2 mL of the sample with 8 mL of PBS, filtering the non-EV material with 0.8 μm syringe filters, and performing overnight ultracentrifugation for 18 h, 100,000 g, 4 °C. After resuspending the resulting pellet in 200 µL of PBS, microbiota-derived EVs were isolated similarly to the fecal samples using density-gradient ultracentrifugation. Two negative control samples (sample-free Falcon tubes with 10 mL of PBS) were processed with the amniotic fluid samples for decontamination analysis. This method was successfully used before in an earlier study, where additionally, bacterial proteins were identified [7]. The ratio of bacterial proteins to human proteins in amniotic fluid was 30/3186 [7].

### EV characterization

EVs were imaged via TEM using a Tecnai G2 Spirit (FEI Company, Hillsboro, OR, USA) and JEM-120i microscope (JEOL Ltd., Japan). Nanoparticle size and concentration were measured by NTA using a NanoSight NS300 (Malvern Panalytical, Malvern, UK). Negative staining for the TEM was performed in the Biocenter Oulu Electron Microscopy Core Facility at the University of Oulu. For the NTA analysis, the samples were diluted to 1:50 or 1:100. The dilution was prepared based on the number of particles seen on screen, with approximately 20–100 particles yielding accurate results. Four 1-min videos were recorded for each sample, from which particle sizes and concentrations were calculated. The results were calculated as means for every 1-min recording for every sample. We compared mean concentration results based on the weight group of each sample for both feces and amniotic fluid.

### RNA isolation

After EV isolation, we isolated RNA from the EV samples using an exoRNeasy serum/plasma midi kit (Qiagen, Hilden, Germany), following the manufacturer’s instructions. Briefly, 10 µL of purified EV and 90 µL of PBS were mixed, Qiazol was added, and the sample was incubated at room temperature. Then chloroform was added, and the sample was incubated again at room temperature and subsequently centrifuged at 12,000 g for 15 min at 4 °C. The upper phase, containing the RNA, was transferred to a new tube, 96% EtOH was added, and the mixture was transferred to an RNeasy MinElute spin column, after which the sample was bound to the column and washed with buffers by centrifuging at 12,000 g at 4 °C. Finally, the purified RNA was eluted in 14 µL of nuclease-free water by centrifuging at 14,000 g and stored at − 80 °C.

### Conversion to cDNA, PCR, and 16 S rRNA gene sequencing

Conversion to cDNA, PCR, and 16 S rRNA gene sequencing were performed by the Biocenter Oulu Sequencing Unit at the University of Oulu. The RT-PCR reaction utilized an iScript cDNA synthesis kit (Bio-Rad, Hercules, CA, USA), and the manufacturer’s instructions were followed. We mixed 10 µL of RNA with 2 µL (0.5 µM) of 16 S rRNA primer 515 F (5′-GTGCCAGCMGCCGCGGTAA-3′) and 1x iScript reaction mix, yielding 20 µL per reaction. The protocol was as follows: 5 min at 25 °C, 20 min at 46 °C, and 1 min at 95 °C.

The samples converted to cDNA were subjected to PCR to amplify the V4–V5 variable region of the 16 S rRNA gene. The primers 519 F (5′-CAGCMGCCCGCGGTAATWC-3′) and 926R (5′-CCGTCAATTCCTTTRAGTTT-3′) were used for the reaction, with the primer 519 F containing a 30-bp Ion Torrent adapter sequence at the beginning and nucleotide linker A between the primer and a unique barcode for each sample. The 926R primer began with the Ion Torrent adapter sequence trP1. The PCR reaction for 30 µL of one sample included 5 µL of cDNA, a primer concentration of 0.75 µM, and 1x Phusion Flash high-fidelity master mix (Thermo Fisher Scientific). The PCR reaction included initialization for 3 min at 98 °C; 32 cycles of 10 s at 98 °C, 10 s at 64 °C, and 30 s at 72 °C; and a final 5-min elongation at 72 °C. The success of the PCR was confirmed with an Agilent Bioanalyzer (Agilent Technologies, Santa Clara, CA, USA).

The PCR products were purified with an Agencourt AMPure XP PCR purification system (Agilent Technologies) and quantified with the Agilent Bioanalyzer using a DNA 1000 analysis kit (Agilent Technologies). The samples were then pooled, purified again, and quality-checked with the Agilent Bioanalyzer before quantification using a Quant-iT™ PicoGreen™ dsDNA assay kit (Thermo Fisher Scientific). For library preparation, a Ligation Sequencing Kit V14 (Oxford Nanopore Technologies, Oxford, UK) was used, with minor modifications to the manufacturer’s instructions. In brief, 200 fmol of the sample pool were treated with an NEBNext Ultra II end repair/dA-tailing module (New England Biolabs, Ipswich, MA, USA), with an incubation period of 30 min. The end-repaired DNA was treated with AMPure XP beads and measured using a PicoGreen™ dsDNA assay kit. Nanopore ligation adapters were attached with T4 DNA ligase via incubation at room temperature for 30 min before further purifying the pool. The library was eluted in 20 µL of elution buffer, and its concentration was measured with a PicoGreen™ dsDNA assay kit.

Oxford Nanopore’s MinION Mk1C was used for sequencing, with an R10.4.1 flow cell. Twenty fmol of the library were mixed with sequencing buffer and library beads and loaded onto a primed flow cell. The sequencing was run in fast basecalling mode for 25 h. Raw POD5 files were transferred and re-basecalled into FASTQ files with Dorado (Version 0.7.2) in sup basecalling mode, with a custom minimum Phred quality score of 18. The sequences have been submitted to Genbank with accession number PRJNA1235131 [17]. 

### Sequence preprocessing and analysis

The sample sequences were preprocessed using QIIME2 (Version 2024.2-amplicon) [18]. First, primer adapters were removed from the sequences using cutadapt (Version 4.6) [19]. Reverse reads were reverse-complemented using Seqtk (Version 1.4; https://github.com/lh3/seqtk) and merged with forward reads. Reads were demultiplexed with cutadapt and trimmed to ensure that the primers were removed with an error rate of 0. Vsearch (Version 2024.2.0) [20] was used to dereplicate sequences and create a feature table, after which operational taxonomic units (OTUs) were picked using the closed-reference strategy and the SILVA database (Version 138) [21] with QIIME2-provided 16 S SSURef NR99 full-length sequences prepared using RESCRIPt [22], with an identity of 97% during clustering. Chimeric reads were identified using vsearch and removed from the feature table and representative sequences along with singletons. The SILVA classifier was trained using the SSURef NR99 full-length taxonomy file provided by QIIME2. Contaminant reads were identified and removed using the decontam package (Version 1.24.0) [23] in R. During contaminant read removal, we calculated the initial library size for each individual sample (Additional File: Fig. S1a). A prevalence-based method with normalization was used in which the abundance of the features is compared in both true and negative control samples, and if the abundance in the negative controls exceeds an assigned threshold (here 0.5), the feature is considered a contaminant (Additional File: Fig. S1b). We filtered out contaminant reads from all our samples, after which we filtered out samples with fewer than 1,000 reads and two features from the analyses, yielding the final sample list (Additional File: Table S1). The taxonomic composition of negative control samples can be observed in Additional File: Fig. S2.

After preprocessing, we performed bacterial diversity and community analyses. For the alpha and beta diversity analyses, we created separate rarefaction curves for the amniotic fluid and fecal samples to estimate the sampling depth at which diversity would likely be represented accurately. The samples were rarefied to an even sampling depth of 5,601 for amniotic fluid and 10,166 for feces. We performed alpha (within-sample) diversity analysis using the Shannon Index and number of observed features. Beta (between-sample) diversity was visualized with principal coordinates analysis (PCoA) using Bray–Curtis dissimilarity. For the community analysis, we calculated the relative abundances of the phyla and genera secreting microbiota-derived EVs. All figures were drawn using R (Version 4.4.1) [24] and the following packages: ggplot2 (Version 3.5.1) [25], qiime2R (Version 0.99.6) [26], tidyverse (Version 2.0.0), reshape2 (Version 1.4.4), plyr (Version 1.8.9), scales (Version 1.3.0) and readr (Version 2.1.5). The code used for this analysis has been uploaded to Zenodo with DOI: 10.5281/zenodo.15119367 [27]. 

### Statistical analysis

We compared the diversity and microbial compositions of different weight groups: lean women, those with overweight, and those with obesity. In addition, comparisons were made between those who were diagnosed with GDM during prenatal care according to national guidelines and those who were not diagnosed with GDM. The statistical analysis for GDM was adjusted for weight. Furthermore, comparisons were made based on weight gain during pregnancy: The groups were < 15 kg and ≥ 15 kg of weight gain during pregnancy. Finally, comparisons were based on whether the women received antibiotics during pregnancy or delivery, whether they had asthma or allergies, and whether they were smoking during pregnancy or not. Samples with missing data were excluded from the statistical analyses. The statistical significance of the NTA and alpha diversity analyses was measured using the Kruskal–Wallis H test with adjusted p-values via the Benjamini–Hochberg method. For the beta diversity analysis, we used permutational multivariate analysis of variance (PERMANOVA) with 999 permutations. For the community analyses, we performed differential abundance analysis using Analysis of Compositions of Microbiomes with Bias Correction (ANCOM-BC) [28] with false discovery rate (FDR)-adjusted p-values. A p-value < 0.05 was considered statistically significant for all analyses.

## Results

### Study cohort

This study included 60 pregnant women. In the first trimester of pregnancy, 20 women (33%) had normal body mass indices (BMI < 25), 27 (45%) had overweight (BMI 25–30), and 13 (22%) had obesity (BMI > 30) (Table 1) [29]. Less than half received antibiotics during pregnancy, mostly consisting of oral β-lactams. Over half of the women received antibiotics during delivery, mostly intravenous penicillin, mainly due to C-section delivery. None of the women had severe underlying medical conditions. In total, 60 fecal samples and 18 amniotic fluid samples were collected, as amniotic fluid samples were only obtained from women undergoing elective C-sections. Of the 60 women, 26 (43%) delivered via C-section.

**Table 1:** Clinical characteristics of the participating women

Clinical characteristics	Feces (*N* = 60)	Amniotic fluid (*N* = 18)
Age (years), mean (SD)	32 (5.6)	32 (4.4)
Body mass index during first trimester, mean (SD)	27 (4.4)	28 (4.6)
Weight group during first trimester		
Lean (BMI < 25) N (%)	20 (33)	6 (33)
Overweight (BMI 25–30) N (%)	27 (45)	5 (28)
Obesity (BMI > 30) N (%)	13 (22)	7 (39)
Duration of pregnancy (gestational weeks) Mean (SD)	40 (1.1)	40 (1.0)
C-section delivery N (%)^a^	26 (43)	18 (100)
Antibiotics during pregnancy N (%)^b^	18 (30)	8 (44)
Antibiotics during delivery N (%)^c, d^	37 (62)	17 (94)
Smoking during pregnancy N (%)^e^	6 (10)	2 (11)
Underlying medical conditions		
Allergies N (%)^f^	17 (28)	4 (22)
Asthma N (%)	5 (8.3)	3 (17)
Inflammatory bowel disease N (%)	1 (1.6)	0 (0)
Gestational diabetes N (%)	19 (32)	5 (28)
Fecal sampling time (days) after delivery, mean (SD)^g^	3.9 (5.4)	-

^a^Amniotic fluid samples were exclusively obtained from C-sections

^b^Mostly oral β-lactams

^c^Mostly penicillin

^d^Antibiotics were routinely given to nearly all women during C-section as surgical site infection prophylaxis and based on positive universal screening for group B streptococcus for those having vaginal deliveries

^e^Missing data for one woman

^f^Missing data for two women

^g^Missing data for two women. Two women provided the fecal sample one day before delivery

Characteristics have been provided separately for feces and amniotic fluid. Amniotic fluid samples were donated by the same women donating fecal samples. SD: Standard deviation

### Characterization of extracellular vesicles

**Fig. 1:**
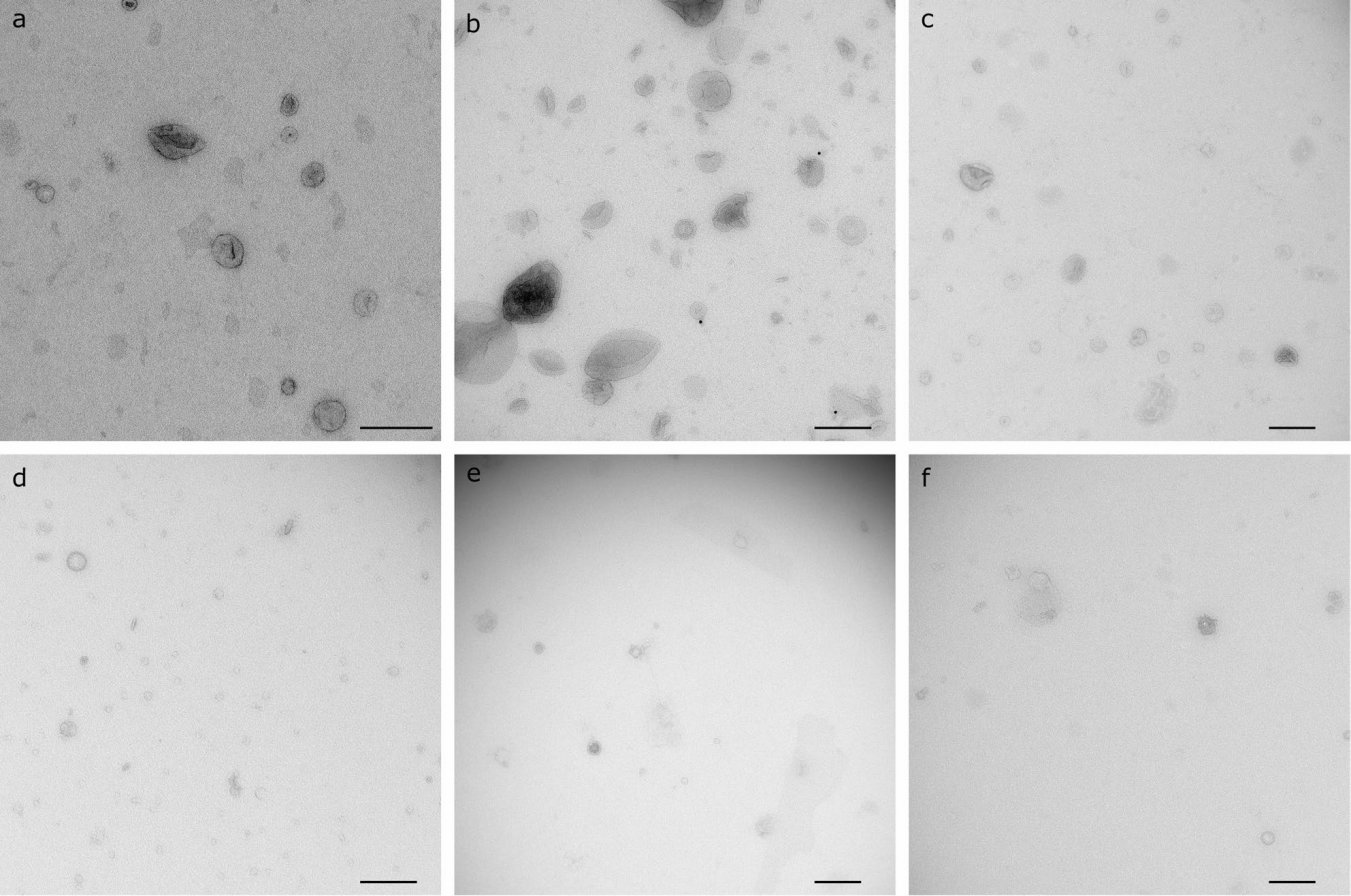
TEM figures for each sample type. **a**: Amniotic fluid, lean. **b**: Amniotic fluid, overweight. **c**: Amniotic fluid, obesity. **d**: Maternal feces, lean. **e**: Maternal feces, overweight. **f**: Maternal feces, obesity. Images were taken with a magnification of 18,500–30,000. The scale bar for each figure is 200 nm

The TEM analysis showed amniotic fluid and fecal samples containing EVs of various sizes, with the EVs obtained from fecal samples being rounder and the EVs from amniotic fluid being more heterogenous in shape (Fig. 1, Additional File: Fig. S3).

**Fig. 2:**
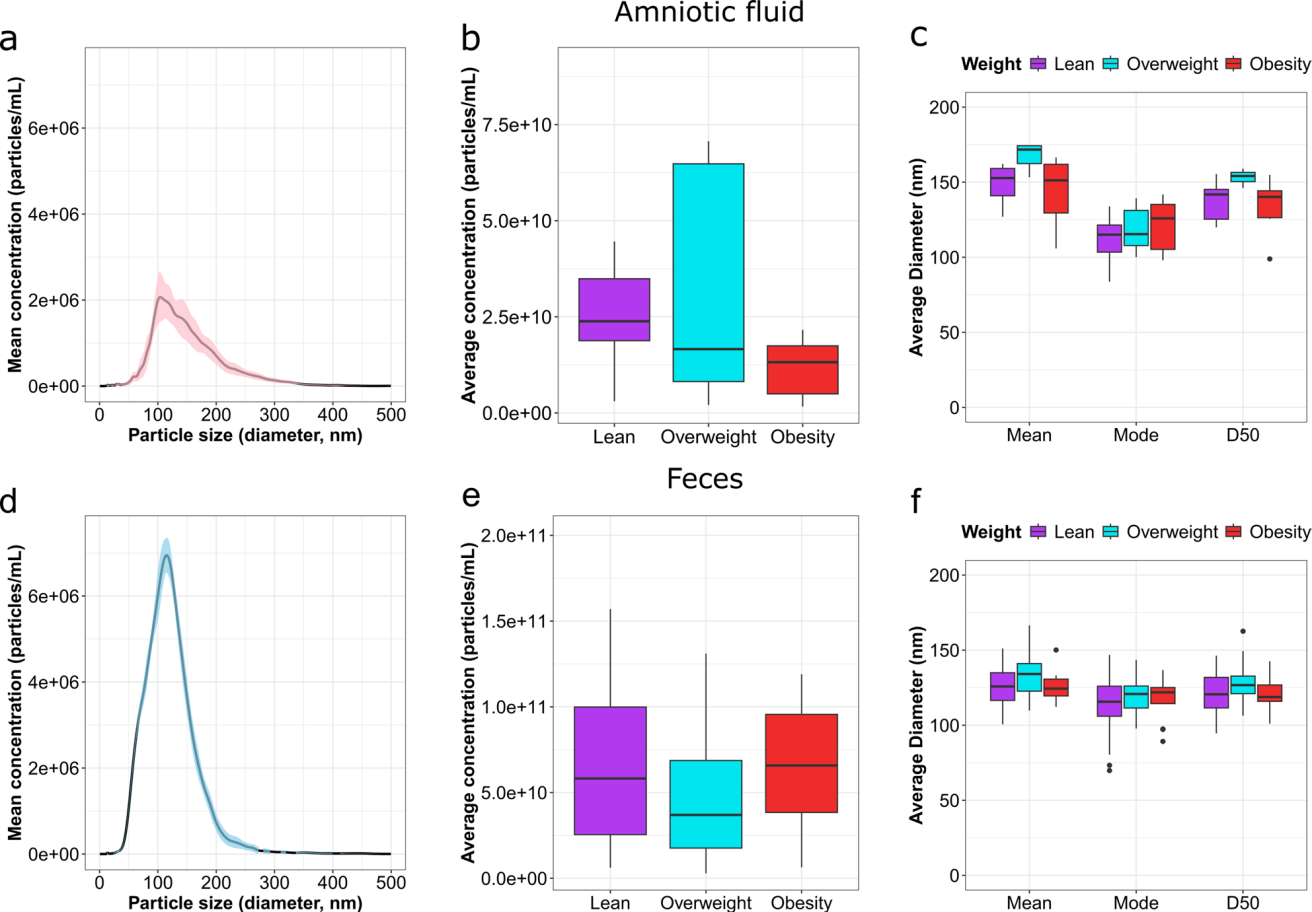
Nanoparticle tracking analysis results for amniotic fluid and feces based on weight. **a**: Average concentration (particles/mL) per particle size (diameter, nm) of amniotic fluid samples. **b**: Average concentration (particles/mL) of amniotic fluid samples. **c**: Average diameter (nm) of amniotic fluid samples. **d**: Average concentration (particles/mL) per particle size (diameter, nm) of fecal samples. **e**: Average concentration (particles/mL) of fecal samples. **f**: Average diameter (nm) of fecal samples. In the size analysis, mean, mode, and D50 values have been calculated along the x-axis. Statistically significant comparisons have been marked in the figures, with an asterisk (*) indicating a p-value < 0.05.

NTA showed that the particle sizes across amniotic fluid and fecal samples were mostly 200 nm and below (Fig. 2a, d), which is a typical size for microbiota-derived EVs [1]. The differences in average nanoparticle concentrations of the lean, overweight, and obesity groups (2.52, 3.25, and 1.16 × 10^10^ particles/mL respectively) were not significantly different in amniotic fluid (Fig. 2b). Similarly, no differences were observed for fecal samples (6.36, 4.36, and 6.5 × 10^10^ particles/mL for the lean, overweight, and obesity groups respectively; see Fig. 2e). Nanoparticle size did not significantly differ based on weight group in the amniotic fluid or fecal samples (Fig. 2c, f).

### Microbiota-derived extracellular vesicles in amniotic fluid samples according to maternal weight

After preprocessing the sequence data to only retain high-quality data (see Methods), 17 amniotic fluid samples from six women who were lean, four with overweight, and seven with obesity were used for the microbial community analysis.

**Fig. 3:**
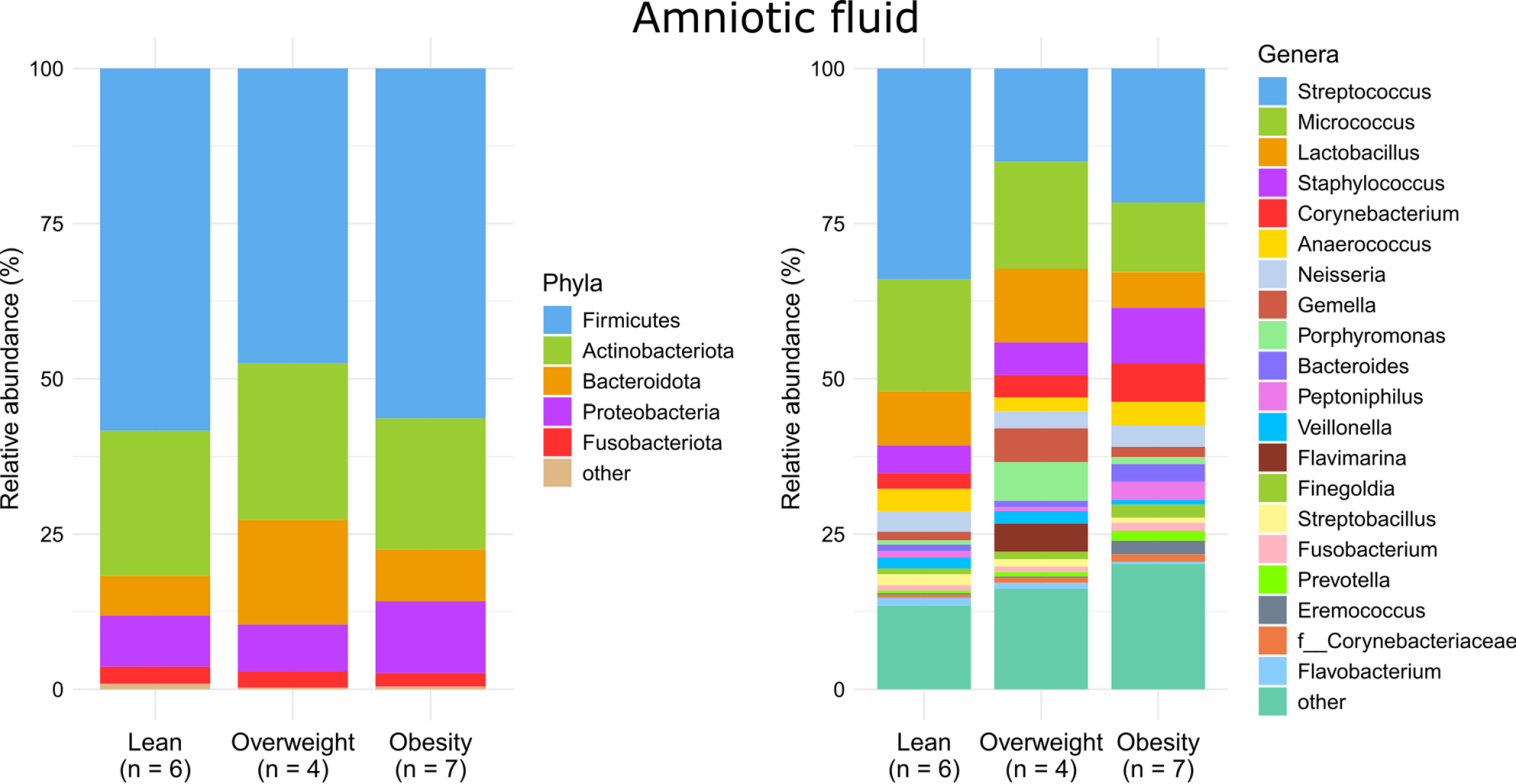
Relative abundance of the origins of microbiota-derived EVs in amniotic fluid samples based on weight group. The five most abundant phyla and 20 most abundant genera have been listed, while the rest of the phyla and genera have been bundled into the “other” group

In the amniotic fluid samples, the most common microbiota-derived EV sources on the phylum level were Firmicutes, Actinobacteriota, Bacteroidota, and Proteobacteria. On the genus level, the most common microbiota-derived EV sources were *Streptococcus*, *Micrococcus*, *Lactobacillus*, and *Staphylococcus* (see Fig. 3; Additional File: Table S2; Additional File: Fig. S4).

ANCOM-BC analysis of the differential abundance of the microbiota-derived EVs in the weight groups was performed (Table 2). Phylum level analysis did not show differences between the weight groups. Genus level analysis showed that *Brevundimonas* was depleted in the overweight group compared to the lean group, and *Bergeyella*, *Bradyrhizobium*, *Alloprevotella*, *Granulicatella*, and *Chryseobacterium* were depleted in the obesity group compared to the lean group.

**Table 2:** Differential abundance of microbiota-derived EV-secreting bacteria in different weight groups using ANCOM-BC

Amniotic fluid										
Comparison against lean		Genus	LFC (comparison)			W		*p*-value		Enriched or depleted in comparison to lean
Overweight		*Brevundimonas*	-2.3			-4.1		0.008		Depleted
Obesity		*Bergeyella*	-3.3			-5.0		< 0.001		Depleted
		*Bradyrhizobium*	-1.9			-4.4		0.001		Depleted
		*Alloprevotella*	-2.6			-3.9		0.007		Depleted
		*Granulicatella*	-0.42			-1.4		0.02		Depleted
		*Chryseobacterium*	-3.1			-3.6		0.02		Depleted

**Feces**										
**Comparison against lean**	**Phylum**			**LFC (obesity)**	**W (obesity)**		**p-value**		**Enriched or depleted in comparison to lean**	
Obesity	Fusobacteriota			-1.6	-3.1		0.03		Depleted	

**Comparison against lean**	**Genus**			**LFC (obesity)**	**W (obesity)**		**p-value**		**Enriched or depleted in comparison to lean**	
Obesity	*Chryseobacterium*			1.9	4.2		0.004		Enriched	
	*Clostridia UCG-014*			-1.5	-4.2		0.004		Depleted	

Amniotic fluid results have been listed above and fecal samples below. LFC: log-fold change. W: log-fold change divided by standard error

### Microbiota-derived extracellular vesicles in maternal fecal samples according to maternal weight

After preprocessing the sequence data to only retain high-quality data (see Methods), we were left with 56 maternal fecal samples, of which 20 were from women who were lean, 25 from women with overweight, and 11 from women with obesity. The weight of the starting material showed no significant differences in microbiota-derived EV sources when grouping samples based on whether we had less than 500 mg (*n* = 10), between 500 and 1000 mg (*n* = 14), or more than 1000 mg (*n* = 23) of fecal material for isolation (Additional File: Table S3).

**Fig. 4:**
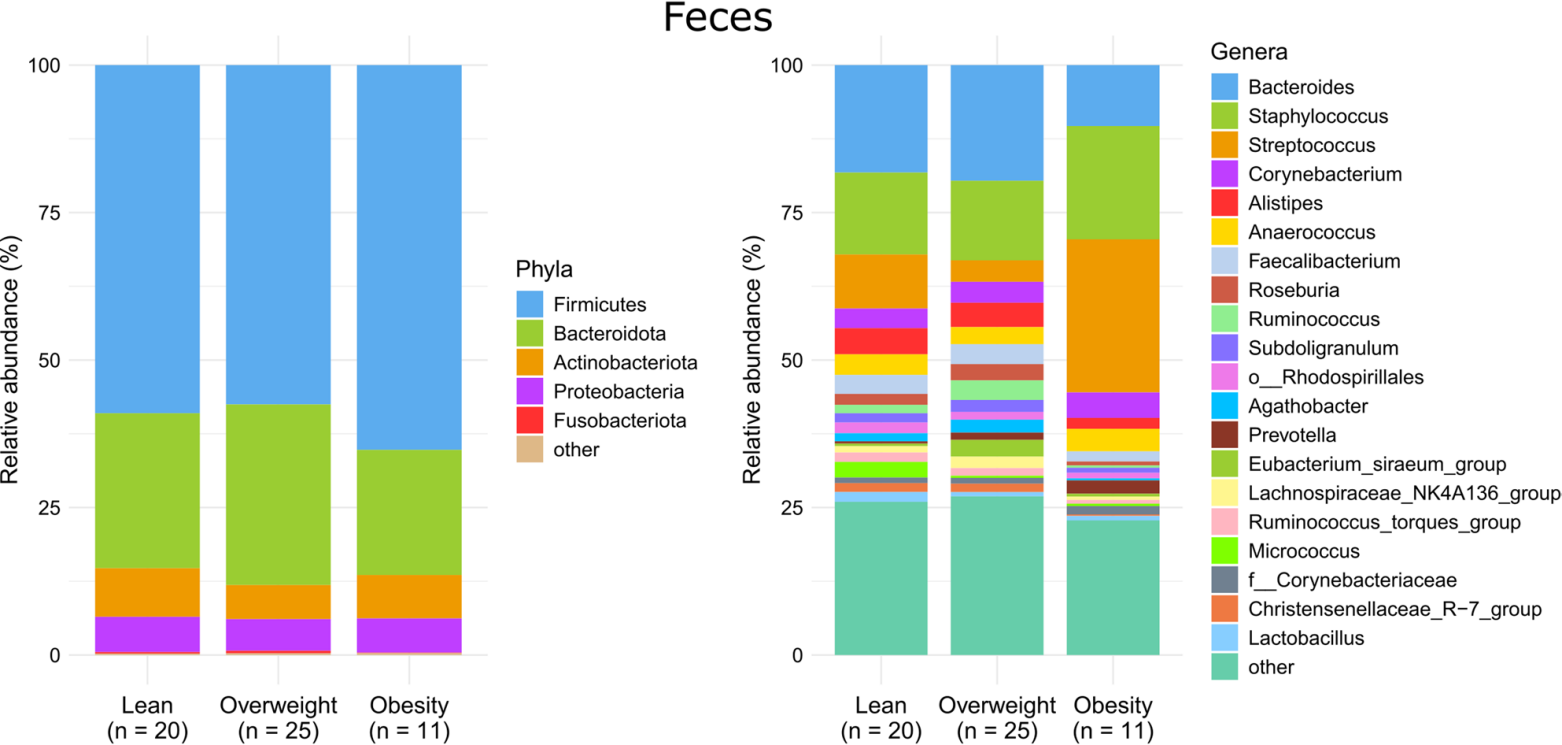
Relative abundance of the origins of microbiota-derived EVs in fecal samples based on weight group. The five most abundant phyla and 20 most abundant genera have been listed, while the rest of the phyla and genera have been bundled into the “other” group

In the fecal samples, the most common microbiota-derived EV sources at the phylum level were Firmicutes, Bacteroidota, Actinobacteriota, and Proteobacteria and at the genus level *Bacteroides*,* Staphylococcus*, *Streptococcus*, and *Corynebacterium* (see Fig. 4; Additional File: Table S4; Additional File: Fig. 5).

ANCOM-BC analysis showed significant differences between the lean and obesity groups: in the obesity group in comparison to the lean group, the phylum Fusobacteriota and genus *Clostridia UCG-014* were depleted, while the genus *Chryseobacterium* was enriched (Table 2).

### Microbial diversity of extracellular vesicles according to maternal weight

**Fig. 5:**
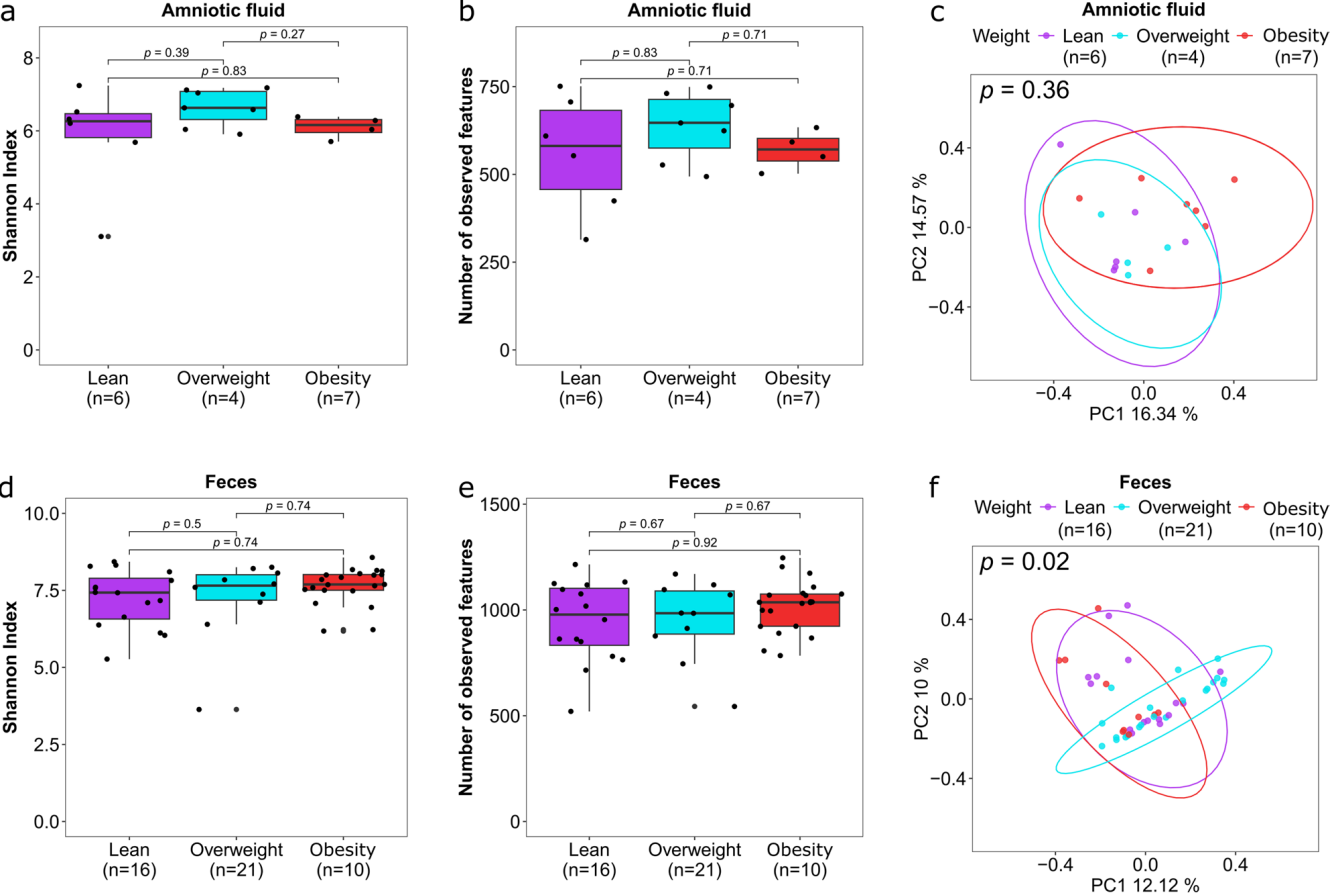
Weight group -based diversity analyses of the microbiota-derived EV origins in amniotic fluid and feces. Shannon Index (a, d) and observed features (b, e) were used as alpha diversity metrics. Beta diversity was depicted as a PCoA plot using Bray–Curtis dissimilarity (c, f). Above, amniotic fluid samples (a–c) are presented, and below, fecal samples (d–f) are presented. Sample numbers for the amniotic fluid samples: lean = 6, overweight = 4 and obesity = 7. Sample numbers for the fecal samples: lean = 16, overweight = 21 and obesity = 10

After rarefication, 17 amniotic fluid and 47 fecal samples were used for diversity analyses of the microbiota-derived EVs. Based on maternal weight, neither the amniotic fluid nor fecal samples showed significant differences in alpha diversity, i.e., within sample diversity (see Fig. 5; Additional File: Table S5).

Regarding beta diversity, i.e., between samples diversity, the women with overweight and women with obesity differed in terms of microbiota-derived EVs in their feces (see Fig. 5; Additional File: Table S5). No other significant differences were found in the amniotic fluid or fecal samples.

### Microbiota-derived extracellular vesicles according to gestational diabetes status

Of the 56 women for whom we had fecal samples and proper sequencing results, 15 (27%) had GDM. Of the 17 women for whom we had amniotic fluid samples and proper sequencing results, five (28%) had GDM.

There was a small difference in the bacterial sources of microbiota-derived EVs in women with GDM and those without based on ANCOM-BC analysis. The genus *Burkholderia-Caballeronia-Paraburkholderia* was enriched in the amniotic fluid collected from women with GDM as compared to those without. There were no differentially abundant taxa in fecal samples based on the GDM status of the woman (Table 3).

**Table 3:** GDM and weight gain -based differential abundance using ANCOM-BC

ANCOM-BC results according to GDM status					
Sample group	Phylum	LFC (yes GDM)	W (yes GDM)	p-value	Enriched or depleted in comparison to no GDM
Amniotic fluid	none				
Feces	none				
Sample group	Genus	LFC (yes GDM)	W (yes GDM)	p-value	Enriched or depleted in comparison to no GDM
Amniotic fluid	*Burkholderia-Caballeronia-Paraburkholderia*	1.1	3.8	0.03	Enriched
Feces	none				

**ANCOM-BC results according to weight gain during pregnancy**					
Sample group	Phylum	LFC (> 15 kg)	W (> 15 kg)	p-value	Enriched or depleted in comparison to < 15 kg
Amniotic fluid	none				
Feces	Fusobacteriota	1.4	3.3	0.013	Enriched
Sample group	Genus	LFC (> 15 kg)	W (> 15 kg)	p-value	Enriched or depleted in comparison to < 15 kg
Amniotic fluid	*Brachybacterium*	-2.5	-4.0	0.015	Depleted
	*Capnocytophaga*	-2.6	-3.8	0.016	Depleted
Feces	none				

Tests were performed on GDM status and weight gain during pregnancy separately for amniotic fluid and feces. LFC: log-fold change. W: Log fold change divided by standard error

The diversity analyses showed no significant differences in either the amniotic fluid or feces based on maternal GDM status (Table 4).

**Table 4:** Diversity results based on gestational diabetes and weight gain during pregnancy

Comparison	Shannon Index		Number of observed features		Bray-Curtis dissimilarity	
	H	*p*-value	H	*p*-value	PERMANOVA pseudo-F	*p*-value
Amniotic fluid, GDM	0.013	0.91	0.21	0.65	1.2	0.25
Amniotic fluid, weight gain during pregnancy	0.15	0.7	0.23	0.63	0.83	0.74
Feces, GDM	0.35	0.55	0.38	0.54	0.93	0.55
Feces, weight gain during pregnancy	1.22	0.27	2.1	0.15	0.87	0.68

Shannon Index, number of observed features, and Bray-Curtis dissimilarity was measured. H = Kruskal–Wallis H

### Microbiota-derived extracellular vesicles according to weight gain during pregnancy

Of the mothers who gave amniotic fluid samples, nine gained less than 15 kg during pregnancy, while eight gained 15 kg or more. Of the mothers who gave fecal samples, 24 gained less than 15 kg, while 23 gained 15 kg or more.

In the women who gained more than 15 kg during pregnancy, the phylum Fusobacteriota was enriched as a source of bacterial extracellular vesicles in feces. At the genus level, *Brachybacterium* and *Capnocytophaga* were depleted in the amniotic fluid samples of those who gained 15 kg or more during pregnancy (Table 3). There were no differences in the diversity of microbiota-derived EV-secreting bacteria based on weight gained during pregnancy (Table 4).

### Microbiota-derived extracellular vesicles according to use of antibiotics and other health factors during pregnancy

We explored the effects of the use of antibiotics during pregnancy and delivery on microbiota-derived EV secretion rates of pregnant women. During pregnancy, women were mainly administered oral β-lactams, and during delivery, intravenous penicillin. In fecal samples, the Shannon Index and the number of observed features were significantly lower (*p* = 0.009 and 0.035, respectively) in women who had received antibiotics during pregnancy compared to women who had not (Table 5). However, this effect was not observed when samples exposed to antibiotics during delivery were removed from the analysis. Amniotic fluid samples showed no differences based on antibiotic exposure during pregnancy. Exposure to antibiotics during delivery did not significantly affect the diversity of microbiota-derived EV-secreting bacteria in stool or amniotic fluid.

We also explored the effects of other background information of pregnant women on the microbiota-derived EV-secretion rates, including allergies, asthma, and smoking during pregnancy. The effects of inflammatory bowel disease were not explored due to only one woman being diagnosed. No significant differences in diversity were observed based on any of these factors in amniotic fluid or stool (Table 5).

**Table 5:** Diversity results based on other health factors during pregnancy

Comparison	Shannon Index		Number of observed features		Bray-Curtis dissimilarity	
	H	*p*-value	H	*p*-value	PERMANOVA pseudo-F	*p*-value
Amniotic fluid, antibiotics during pregnancy (all samples) (no = 10, yes = 7)	0.04	0.85	0.04	0.85	0.91	0.62
Amniotic fluid, asthma (no = 14, yes = 3)	0.25	0.61	0.78	0.38	1.0	0.42
Amniotic fluid, allergies (no = 12, yes = 4)	0.94	0.33	0.53	0.47	0.62	0.97
Amniotic fluid, smoking during pregnancy (no = 15, yes = 2)	1.4	0.23	1.4	0.23	0.62	0.97
Amniotic fluid, antibiotics during delivery (no = 1, yes = 16)	0.38	0.54	1.5	0.22	1.2	0.34
Feces, antibiotics during pregnancy (all samples) (no = 31, yes = 16)	6.8	0.009	4.5	0.035	1.33	0.076
Feces, antibiotics during pregnancy (samples with no intrapartum antibiotics) (no = 11, yes = 3)	3.8	0.052	3.2	0.073	1.3	0.083
Feces, asthma (no = 43, yes = 4)	2.81	0.093	1.2	0.27	1.1	0.31
Feces, allergies (no = 33, yes = 12)	0.26	0.61	0.78	0.45	1.4	0.20
Feces, smoking during pregnancy (no = 42, yes = 4)	2.8	0.24	0.24	0.63	1.4	0.20
Feces, antibiotics during delivery (no = 14, yes = 33)	1.0	0.31	1.7	0.19	1.3	0.059

Shannon Index, number of observed features, and Bray-Curtis dissimilarity was measured. H = Kruskal–Wallis H

## Discussion

In the present study, we investigated maternal microbiota-derived EVs, which have previously been shown to reach the fetus during pregnancy [7] and form a distinct entity from the gut microbiota [3]. Our findings show that maternal factors are associated with the compositions of microbiota-derived extracellular vesicles both in amniotic fluid and in maternal feces during pregnancy.

Host-derived EVs, produced by maternal cells, have been extensively studied [30, 31]. Maternal microbiota-derived EVs, however, have been scarcely characterized [7, 32, 33]. There are several studies, however, on the composition of maternal gut microbiota during pregnancy. Gut microbiota changes during pregnancy, mostly due to altered food intake, gradual weight gain, and metabolic changes [34]. Pre-pregnancy weight and weight gain during pregnancy also alter the gut microbiota of pregnant women [35]. A higher concentration of energy and glucose metabolites have been associated with microbiota changes in pregnant women with obesity in early pregnancy [36]. An earlier study by Koren et al. found that particularly women with obesity or GDM had lowered gut microbial diversity during the third trimester [37]. Surprisingly few studies have explored the effects of antibiotics during pregnancy on the maternal microbiota, as the focus is often on the infant rather than maternal health [38]. A study by Stokholm et al. concluded that antibiotics during pregnancy caused an increase of *Staphylococcus* in the vaginal microbiota of pregnant women [39]. Our study is one of the first to consider microbiota-derived EVs in the fetal environment and maternal factors [7].

Our results on microbiota-derived EV sources in the maternal microbiota align with previous microbiota studies showing that obesity has significant associations with the gut microbiota [40]. Here, microbiota-derived EVs are secreted by common gut commensals such as *Bacteroides*, *Alistipes*, and *Faecalibacterium* [41], as well as other typically less common genera in the gut, such as *Streptococcus* and *Staphylococcus*. We found *Clostridia UCG-014* to be depleted in women with obesity compared to women who were lean which aligns with previous microbiota findings in obesity [40], although the effect was smaller here. During pregnancy, the body undergoes many changes which are also reflected in the gut microbiota [34]. Reduced gut microbiota diversity throughout pregnancy has been observed not only in those with obesity and GDM but also in lean and healthy women [37]. These changes during pregnancy may mask possible differences in gut microbiota-derived EVs between pregnant women with obesity and those with normal weight.

In the present study, we found amniotic fluid microbiota-derived EVs to be secreted by genera such as *Streptococcus*, *Micrococcus*, *Lactobacillus*, *Staphylococcus*, and *Corynebacterium*, largely aligning with our previous microbiota-derived EV findings in a fetal environment [7]. Here, we found significant differences between microbiota-derived EVs based on maternal weight, specifically the depletion of *Brevundimonas* in women with overweight and the depletion of *Bergeyella*, *Bradyrhizobium*, *Alloprevotella*, *Granulicatella*, and *Chryseobacterium* in women with obesity compared to lean women. Some species of *Brevundimonas* act as pathogens at various body sites [42], *Alloprevotella* and *Granulicatella* are common gut commensals, and *Chryseobacterium* and *Bergeyella* act as potential pathogens in the human microbiota. As these genera were found only in low abundances in the present study, their clinical and biological effects remain uncertain. *Bradyrhizobium*, a common laboratory contaminant in microbiota studies [43], is not likely to be a relevant finding, beyond highlighting the difficulties of identifying true findings in microbiota studies. This is true especially for low-biomass samples such as amniotic fluid samples and microbiota-derived EV samples isolated by density-gradient ultracentrifugation [44]. The effect of laboratory and reagent contamination in low-biomass samples especially is a difficult challenge to overcome and requires careful laboratory work and use of technical controls as an additional tool for contamination control [45].

We found a small association between microbiota-derived EVs and the use of antibiotics during pregnancy or delivery in maternal feces, with antibiotic use reducing the alpha diversity of microbiota-derived EV sources. This effect was not observed in amniotic fluid, possibly due to a limited sample size. Interestingly, the use of intrapartum antibiotics did not seem to have observable effects on the microbiota-derived EVs in feces or amniotic fluid. This may have been caused by uneven sample groups as most of the women here received intrapartum antibiotics. The use of antibiotics in general has been known to alter the gut microbiota by reducing bacterial diversity, affecting the metabolic activity of bacteria, and increasing antibiotic resistance genes [46]. However, as pregnancy affects gut microbiota composition by itself, the effects of antibiotics on maternal microbiota may be reduced in comparison to non-pregnant individuals.

The presence of microbiota in the amniotic fluid during normal pregnancy is controversial [47–51]. We have previously shown the presence of microbiota-derived EVs in the amniotic fluid both during healthy human pregnancies and in an animal model [7]. The controversial findings of microbial genetic material in traditionally sterile body compartments could thus be explained by microbiota-derived EVs. The present study suggests lower relative abundance of microbiota-derived EVs from normal human microbiota commensals in the amniotic fluid during pregnancy in women with overweight and obesity. In the future, the functions of the microbiota-derived EVs mentioned here need to be explored with a broader omics approach to understand the cellular and molecular-level interaction between the maternal microbiota and the fetus.

Our finding that maternal factors are associated with differences in microbiota-derived EV composition in the amniotic fluid is clinically important because this may influence fetal development. He et al. found similarities between bacterial findings in newborn infants’ first-pass meconium and amniotic fluid [52], implying that bacterial content, including microbiota-derived EVs may “colonize” the gut of the fetus. Furthermore, we have previously found microbiota-derived EVs of unknown origin in the first-pass meconium of newborns [53]. Previous studies have suggested that the risk of childhood obesity is affected not only by inheritance of maternal microbiota after birth but also by exposure to maternal microbiota during the fetal period [54]. Hypothetically, obesity-associated microbiota-derived EVs in the amniotic fluid could contribute to obesity-associated microbiota transfer during pregnancy by modulating the gut metabolism and immune system of the fetus.

This study has several strengths. While most microbiota-derived EV studies focus on microbiota-derived EVs derived from bacterial cultures [55], we were able to obtain clinical samples in a sterile manner from amniotic fluid and in a standard manner from maternal fecal samples. We were able to use state-of-the-art techniques to characterize microbiota-derived EVs in fecal and amniotic fluid samples by following current guidelines for EV studies [44]. Especially in the case of the fecal samples, the sample size was sufficient to produce reliable results. We used negative controls, approximately 10% of the final sample size, to identify and remove environmental and reagent contamination from the study samples using bioinformatics tools.

This study also has some limitations. The number of amniotic fluid samples was relatively small, which limits statistical power especially when subgrouping the samples further. Thus, a larger sample size is required to further confirm these results. The sample size here is comparable to earlier microbiota-derived EV studies in amniotic fluid, ranging from 26 to 28 [7, 32]. Similarly, the analysis of other background factors, such as the use of antibiotics during pregnancy, may have been affected by limited group sizes in both amniotic fluid and feces when removing samples exposed to antibiotics during delivery. However, the methodology in this study was extensive, and the sample size aligned with previous studies that used similar microbiota-derived EV isolation methods [33, 56, 57]. Furthermore, as only a partial region of the 16 S rRNA gene was sequenced, the taxonomic resolution was insufficient for reliable species level identification, which may be achieved using full-length 16 S rRNA gene sequencing or metagenomics approaches. However, this is still a common practice in the field, which makes our present results comparable to those of previous gut and amniotic fluid microbiota studies. Regarding the EV characterization, NTA and TEM analysis may have contained some microbiota-derived EV-sized host EVs as well. Finally, as this study was observational, further studies are needed to characterize maternal microbiota-derived EVs and understand their biological and clinical significance.

## Conclusions

In conclusion, maternal factors had associations with maternal microbiota-derived EV profiles in the feces and amniotic fluid. The potential effects of maternal microbiota-derived EVs on fetal development warrant further research.

## Supplementary Information

Below is the link to the electronic supplementary material.

Supplementary Material 1

Supplementary Material 2

## Data Availability

Raw sequences were submitted to Genbank with accession number PRJNA1235131[55]. The detailed EV methods have been uploaded to EV-TRACK [14] (EV-TRACK ID: EV250013) [15]. The code for data analysis has been uploaded to Zenodo under DOI: 10.5281/zenodo.15119368 [27].

## Abbreviations

EV: extracellular vesicle
TEM: transmission electron microscopy
NTA: nanoparticle tracking analysis
cDNA: complementary DNA
GDM: gestational diabetes
PBS: Phosphate-buffered saline
PCR: polymerase chain reaction
OTU: operational taxonomic unit
PCoA: principal coordinates analysis
PERMANOVA: permutational multivariate analysis of variance
ANCOM-BC: analysis of compositions of microbiomes with bias correction
FDR: false discovery rate
BMI: body mass index
SD: standard deviation
LFC: Log fold change
