# Supplementary figures and images for: Maternal factors associate with microbiota-derived extracellular vesicle profiles in pregnancy: a clinical cohort study
Source: BMC Med. 2026 May 28;24:352. doi: 10.1186/s12916-026-04960-3 (PMC13255227; doi:10.1186/s12916-026-04960-3)

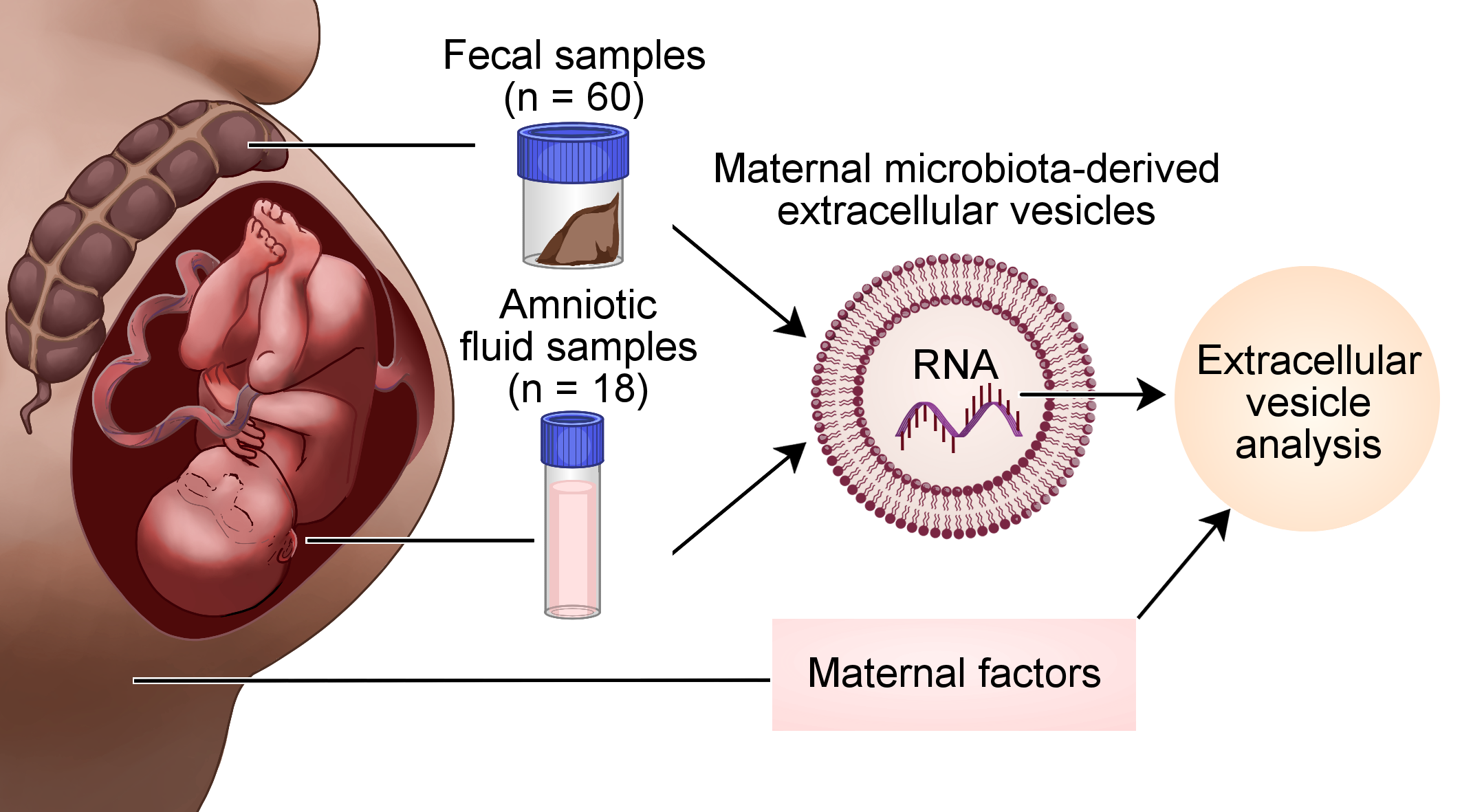

Supplement: Supplementary file 1 — Supplementary Material 1 [file 12916_2026_4960_MOESM1_ESM.png]
