# Supplementary material for: Maternal factors associate with microbiota-derived extracellular vesicle profiles in pregnancy: a clinical cohort study
Source: BMC Med. 2026 May 28;24:352. doi: 10.1186/s12916-026-04960-3 (PMC13255227; doi:10.1186/s12916-026-04960-3)
Supplement: Supplementary file 2 — Supplementary Material 2 [file 12916_2026_4960_MOESM2_ESM.docx]

**Additional files**

**
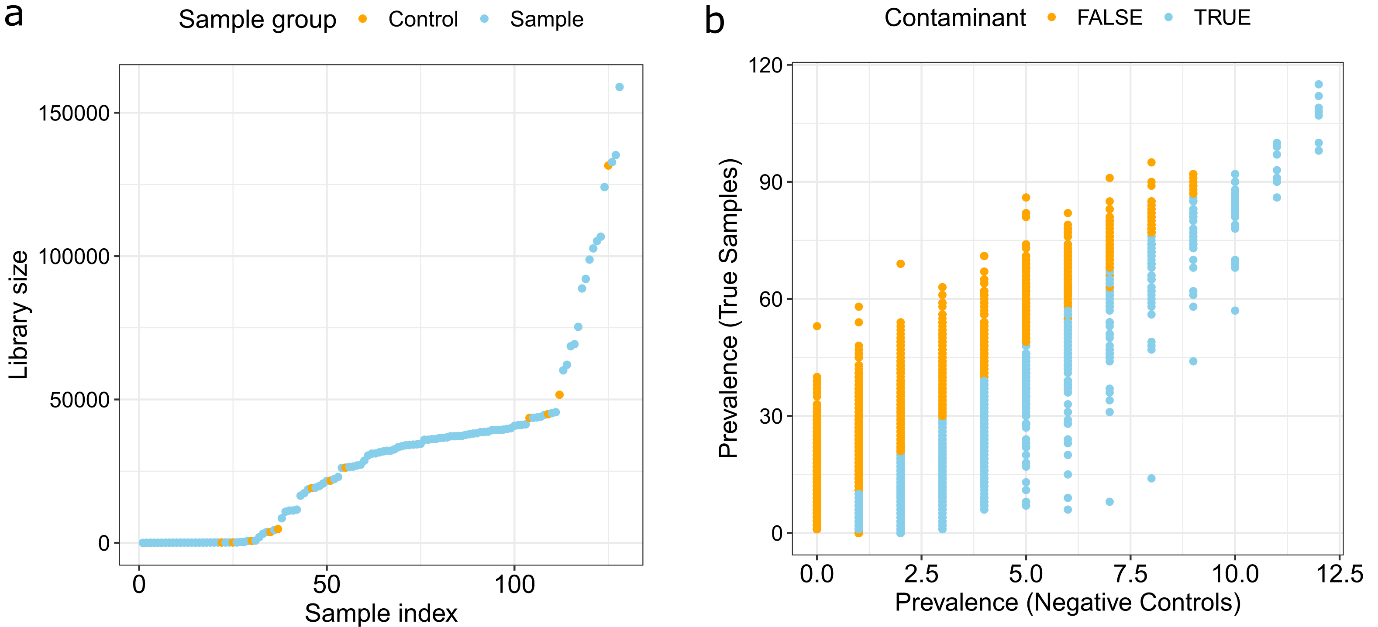
**

**Additional File: Fig. S1. The decontamination process for samples.** a: The original library size for each sample, with true samples (sample) and control samples (control) coded with different colors. The x-axis represents a sample index. b: The prevalence of features in true samples (y-axis) compared to controls (x-axis). The blue color represents those features that have been deemed as contaminants in the data.

**Additional File: Table S1.** **Read counts for all samples examined in the study.** Paired samples have been listed side by side except for the

| Amniotic fluid sample | Frequency | Fecal sample | Frequency | Control sample | Frequency |
| --- | --- | --- | --- | --- | --- |
| A1 | 20,179 | F1 | 29,436 | Ac1 | 21,191 |
| A2 | 6,440 | F2 | 15,617 | Ac2 | 7,002 |
| A3 | 15,258 | F3 | 10,427 | Sc1 | 0 |
| A4 | 26,443 | F4 | 16,480 | Sc2 | 21,953 |
| A5 | 16,154 | F5 | 9,267 | Sc3 | 11,812 |
| A6 | 15,534 | F6 | 15,619 | Sc4 | 11,235 |
| A7 | 15,444 | F7 | 19,618 | Sc5 | 5,935 |
| A8 | 17,007 | F8 | 31,973 | Sc6 | 0 |
| A9 | 10,286 | F9 | 14,859 | PCR_Blank1 | 0 |
| A10 | 15,057 | F10 | 14,786 | PCR_Blank2 | 0 |
| A11 | 7,256 | F11 | 16,490 |  |  |
| A12 | 5,601 | F12 | 17,767 |  |  |
| A13 | 6,962 | F13 | 28,507 |  |  |
| A14 | 33,223 | F14 | 26,115 |  |  |
| A15 | 20,794 | F15 | 15,553 |  |  |
|  |  | F16 | 28,116 |  |  |
| A17 | 12,929 | F17 | 14,187 |  |  |
|  |  | F18 | 15,505 |  |  |
| A19 | 20,347 | F19 | 13,722 |  |  |
|  |  | F20 | 18,288 |  |  |
|  |  | F21 | 2,373 |  |  |
|  |  | F22 | 47,906 |  |  |
|  |  | F23 | 38,091 |  |  |
|  |  | F24 | 69,655 |  |  |
|  |  | F25 | 26,426 |  |  |
|  |  | F26 | 36,449 |  |  |
|  |  | F27 | 3,923 |  |  |
|  |  | F28 | 18,014 |  |  |
|  |  | F29 | 17,902 |  |  |
|  |  | F30 | 21,355 |  |  |
|  |  | F31 | 31,105 |  |  |
|  |  | F32 | 12,977 |  |  |
|  |  | F33 | 79,946 |  |  |
|  |  | F34 | 9,843 |  |  |
|  |  | F35 | 29,337 |  |  |
|  |  | F36 | 9,871 |  |  |
|  |  | F37 | 7,184 |  |  |
|  |  | F38 | 10,878 |  |  |
|  |  | F39 | 5,584 |  |  |
|  |  | F40 | 3,576 |  |  |
|  |  | F41 | 10,166 |  |  |
|  |  | F42 | 1,583 |  |  |
|  |  | F43 | 11,737 |  |  |
|  |  | F44 | 16,213 |  |  |
|  |  | F45 | 15,341 |  |  |
|  |  | F46 | 3,556 |  |  |
|  |  | F47 | 13,308 |  |  |
|  |  | F48 | 16,639 |  |  |
|  |  | F49 | 2,044 |  |  |
|  |  | F50 | 22,924 |  |  |
|  |  | F51 | 5,710 |  |  |
|  |  | F52 | 18,114 |  |  |
|  |  | F53 | 8,860 |  |  |
|  |  | F54 | 12,796 |  |  |
|  |  | F55 | 31,486 |  |  |
|  |  | F56 | 24,854 |  |  |

**
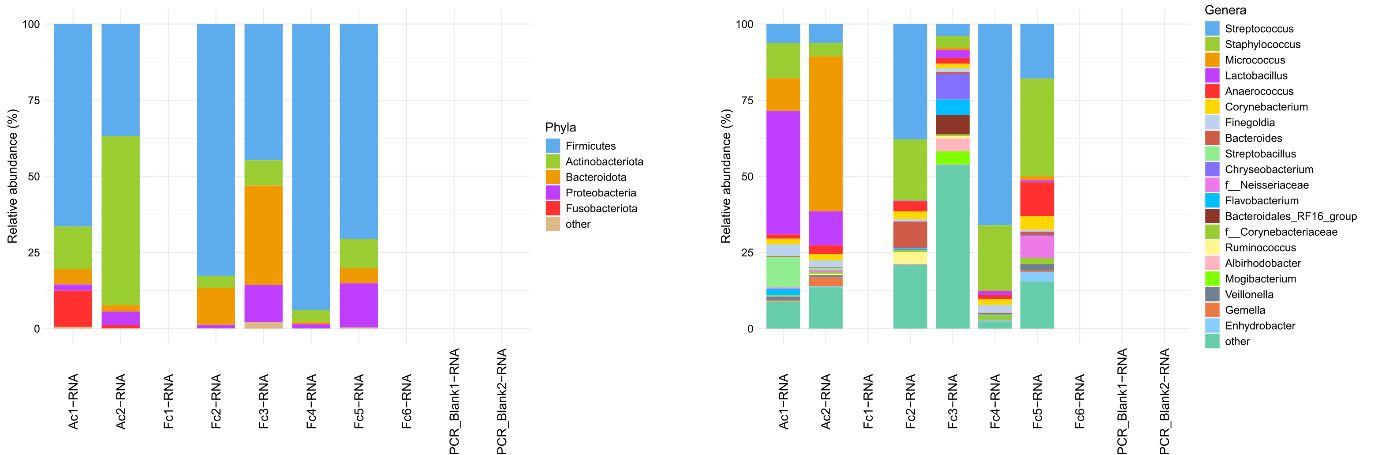
**

**Additional File: Fig. S2. The taxonomic composition of all negative control samples used in the study.** Taxonomic composition of each individual sample is shown in phylum and genus level as relative abundances. Samples with no bars didn’t have enough sequences during preprocessing. The five most abundant phyla and 20 most abundant genera have been listed. while the rest of the phyla and genera have been bundled into the “other” group.

**
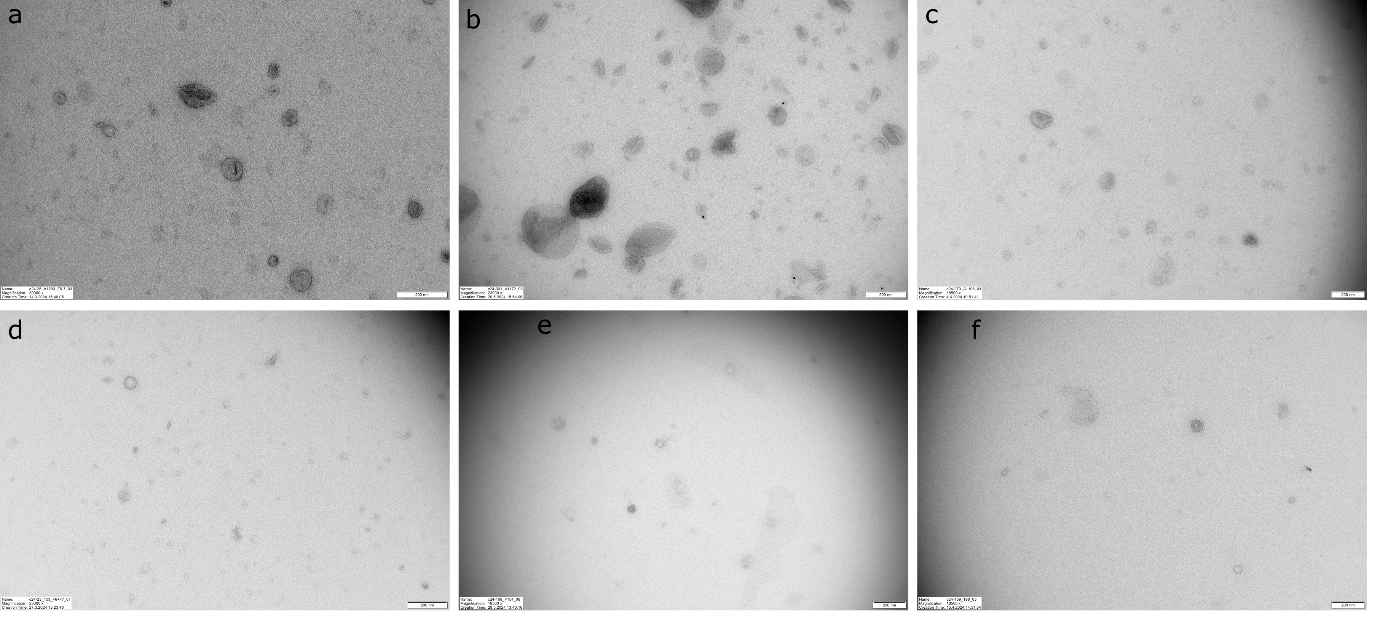
**

**Additional File: Fig. S3. The original TEM images without cropping for each sample type.** a: Amniotic fluid, lean. b: Amniotic fluid, overweight. c: Amniotic fluid, obesity. d: Maternal feces, lean. e: Maternal feces, overweight. f: Maternal feces, obesity.

**Additional File: Table S2.** **The relative abundance of the five most abundant phyla and the 20 most abundant genera in amniotic fluid samples.** Samples have been grouped by weight. Taxa not been identified on genus level have been reported with the lowest taxonomic level available. f: family

| Phylum | Lean (%) | Overweight (%) | Obesity (%) | Genus | Lean (%) | Overweight (%) | Obesity (%) |
| --- | --- | --- | --- | --- | --- | --- | --- |
| Firmicutes | 58 | 48 | 56 | *Streptococcus* | 34 | 15 | 22 |
| Actinobacteriota | 23 | 25 | 21 | *Micrococcus* | 18 | 17 | 11 |
| Bacteroidota | 6.4 | 17 | 8.4 | *Lactobacillus* | 8.7 | 12 | 5.8 |
| Proteobacteria | 8.3 | 7.6 | 12 | *Staphylococcus* | 4.4 | 5.3 | 8.9 |
| Fusobacteriota | 2.8 | 2.6 | 2.2 | *Corynebacterium* | 2.6 | 3.6 | 6.2 |
| other | 0.9 | 0.3 | 0.4 | *Anaerococcus* | 3.7 | 2.2 | 3.8 |
|  |  |  |  | *Neisseria* | 3.3 | 2.8 | 3.4 |
|  |  |  |  | *Gemella* | 1.4 | 5.4 | 1.7 |
|  |  |  |  | *Porphyromonas* | 0.7 | 6.3 | 1.1 |
|  |  |  |  | *Bacteroides* | 1.0 | 1.0 | 2.8 |
|  |  |  |  | *Peptoniphilus* | 1.0 | 0.6 | 2.9 |
|  |  |  |  | *Veillonella* | 1.8 | 2.0 | 0.8 |
|  |  |  |  | *Flavimarina* | 0.0 | 4.5 | 0.0 |
|  |  |  |  | *Finegoldia* | 0.9 | 1.2 | 2.1 |
|  |  |  |  | *Streptobacillus* | 1.8 | 1.2 | 0.8 |
|  |  |  |  | *Fusobacterium* | 0.9 | 0.9 | 1.3 |
|  |  |  |  | *Prevotella* | 0.4 | 0.7 | 1.6 |
|  |  |  |  | *Eremococcus* | 0.3 | 0.2 | 2.2 |
|  |  |  |  | f. Corynebacteriaceae | 0.5 | 0.9 | 1.2 |
|  |  |  |  | *Flavobacterium* | 1.2 | 0.9 | 0.4 |
|  |  |  |  | other | 14 | 16 | 20 |


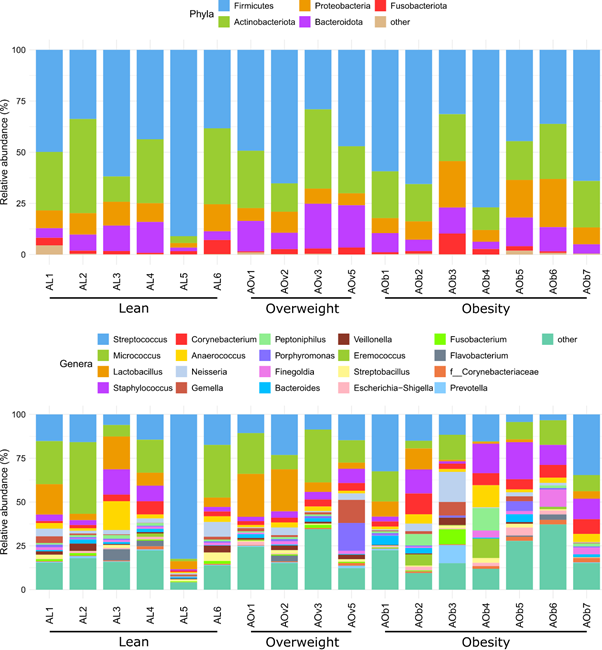


**Additional File: Fig. S4.** **Relative abundance of bacteria secreting microbiota-derived EVs in all amniotic fluid samples at the phylum and genus levels.** The five most abundant phyla and 20 most abundant genera have been listed. while the rest of the phyla and genera have been bundled into the “other” group.

**Additional File: Table S3.** **The diversity analysis of fecal samples based on how much starting material was available for the sample.** 23 samples had 1000 mg or more, 14 samples had between 500 and 1000 mg, and 10 samples had less than 500 mg of starting material for EV isolation. Shannon Index, number of observed features, and Bray-Curtis dissimilarity was measured. H = Kruskal–Wallis H.

|  | Shannon Index | | Number of observed features | | Bray-Curtis dissimilarity | |
| --- | --- | --- | --- | --- | --- | --- |
| Comparison | H | p-value | H | p-value | PERMANOVA pseudo-F | p-value |
| 1000 or more, 500–1000 | 0.283 | 0.784 | 0.542 | 0.462 | 1.015 | 0.661 |
| 1000 or more, less than 500 | 0.075 | 0.784 | 0.812 | 0.462 | 0.875 | 0.661 |
| 500–1000,  less than 500 | 0.415 | 0.784 | 1.814 | 0.462 | 0.949 | 0.661 |

**Additional File: Table S4.** **The relative abundance of the five most abundant phyla and the 20 most abundant genera in fecal samples.** Samples have been grouped by weight. Taxa not been identified on genus level have been reported with the lowest taxonomic level available. o: order. f: family

| Phylum | Lean (%) | Overweight (%) | Obesity (%) | Genus | Lean (%) | Overweight (%) | Obesity (%) |
| --- | --- | --- | --- | --- | --- | --- | --- |
| Firmicutes | 59 | 58 | 65 | *Bacteroides* | 18 | 20 | 10 |
| Bacteroidota | 26 | 31 | 21 | *Staphylococcus* | 14 | 14 | 19 |
| Actinobacteriota | 8.2 | 5.8 | 7.4 | *Streptococcus* | 9.2 | 3.6 | 26 |
| Proteobacteria | 6.0 | 5.4 | 5.8 | *Corynebacterium* | 3.3 | 3.5 | 4.3 |
| Fusobacteriota | 0.3 | 0.4 | 0.1 | *Alistipes* | 4.4 | 4.1 | 1.8 |
| other | 0.2 | 0.3 | 0.3 | *Anaerococcus* | 3.5 | 2.9 | 3.8 |
|  |  |  |  | *Faecalibacterium* | 3.2 | 3.4 | 1.7 |
|  |  |  |  | *Roseburia* | 1.9 | 2.8 | 0.7 |
|  |  |  |  | *Ruminococcus* | 1.4 | 3.3 | 0.4 |
|  |  |  |  | *Subdoligranulum* | 1.6 | 2.0 | 0.9 |
|  |  |  |  | o. Rhodospirillales | 1.8 | 1.3 | 0.9 |
|  |  |  |  | *Agathobacter* | 1.4 | 2.2 | 0.4 |
|  |  |  |  | *Prevotella* | 0.4 | 1.2 | 2.3 |
|  |  |  |  | *Eubacterium siraeum group* | 0.4 | 2.8 | 0.5 |
|  |  |  |  | *Lachnospiraceae NK4A136 group* | 1.1 | 1.9 | 0.6 |
|  |  |  |  | *Ruminococcus torques group* | 1.6 | 1.3 | 0.6 |
|  |  |  |  | *Micrococcus* | 2.6 | 0.4 | 0.4 |
|  |  |  |  | f. Corynebacteriaceae | 1.0 | 1.0 | 1.4 |
|  |  |  |  | *Christensenellaceae R-7 group* | 1.5 | 1.4 | 0.3 |
|  |  |  |  | *Lactobacillus* | 1.7 | 0.7 | 0.8 |
|  |  |  |  | other | 26 | 27 | 23 |


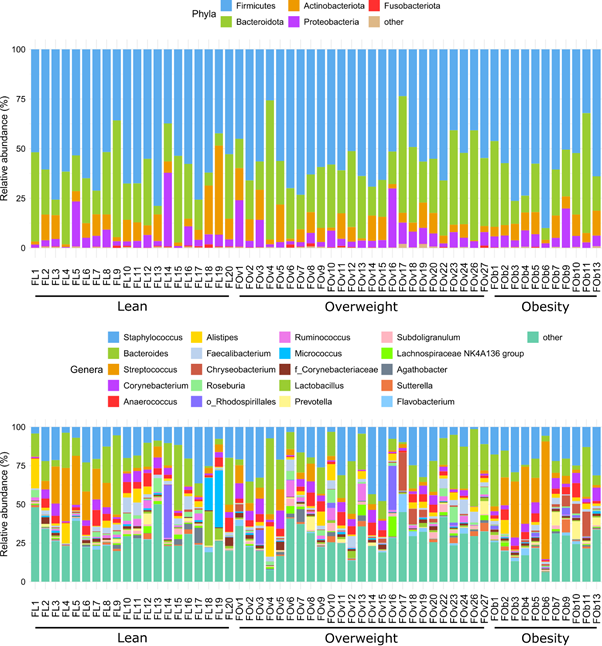


**Additional File: Fig. S5.** **Relative abundance of bacteria secreting microbiota-derived EVs in all fecal samples at the phylum and genus levels.** The five most abundant phyla and 20 most abundant genera have been listed. while the rest of the phyla and genera have been bundled into the “other” group.

**Additional File: Table S5. Pairwise statistical analysis of the diversity results for amniotic fluid and feces based on weight group.** H = Kruskal–Wallis H.

| **Diversity analysis of amniotic fluid** | | | | | | | |
| --- | --- | --- | --- | --- | --- | --- | --- |
| Pairwise comparison | | Shannon Index | | Number of observed features | | Bray-Curtis dissimilarity | |
| Group 1 | Group 2 | H | p-value | H | p-value | PERMANOVA pseudo-F | p-value |
| Lean | Obesity | 1.31 | 0.38 | 0.51 | 0.71 | 1.3 | 0.14 |
| Lean | Overweight | 0.045 | 0.83 | 0.045 | 0.83 | 0.70 | 0.90 |
| Obesity | Overweight | 2.89 | 0.27 | 1.3 | 0.71 | 1.2 | 0.22 |
| **Diversity analysis of feces** | | | | | | | |
| Pairwise comparison | | Shannon Index | | Number of observed features | | Bray-Curtis dissimilarity | |
| Group 1 | Group 2 | H | p-value | H | p-value | PERMANOVA pseudo-F | p-value |
| Lean | Obesity | 0.18 | 0.74 | 0.011 | 0.92 | 1.1 | 0.26 |
| Lean | Overweight | 1.90 | 0.50 | 1.0 | 0.67 | 1.1 | 0.27 |
| Obesity | Overweight | 0.11 | 0.74 | 0.58 | 0.67 | 1.9 | 0.007 |
